# Undifferentiated-type gastric adenocarcinoma: prognostic impact of three histological types

**DOI:** 10.1186/1477-7819-10-254

**Published:** 2012-11-26

**Authors:** Han Hong Lee, Kyo Young Song, Cho Hyun Park, Hae Myung Jeon

**Affiliations:** 1 Division of Gastrointestinal Surgery, Department of Surgery, College of Medicine, The Catholic University of Korea, Seoul, South Korea; 2 Department of Surgery, Uijeongbu St. Mary’s Hospital, College of Medicine, The Catholic University of Korea, 65-1 Gumo-Dong, Uijeongbu-City, Gyenggi-Do, 480-717, South Korea

**Keywords:** Mucinous adenocarcinoma, Signet ring cell carcinoma, Stomach neoplasms, Tubular adenocarcinoma

## Abstract

**Background:**

The prognostic value of the three constituents of undifferentiated-type gastric adenocarcinoma remains unclear. The present study assessed the clinicopathological characteristics and prognosis of undifferentiated-type mucinous adenocarcinoma (uMAC) and signet ring cell carcinoma (SRC) compared with those of poorly differentiated adenocarcinoma (PDAC).

**Methods:**

In total, 1,376 patients with undifferentiated-type gastric adenocarcinoma were included, consisting of 1,002 patients diagnosed with PDAC, 54 with uMAC and 320 with SRC. Clinicopathological factors and survival rates were compared among the three histological types.

**Results:**

Significant differences in the distribution of pathological stages were observed among the groups. Patients with SRC had a significantly better survival rate than those with PDAC or uMAC, in both the all patients including non-curative resected patients and curative-resected groups. In addition, there was significant difference in survival between the PDAC and uMAC groups. Multivariate analysis suggested that age, gender, tumor depth, lymph node metastasis and curability significantly affected survival. Histological type was not an independent prognostic factor. There was no significant difference in the pattern of recurrence among the three groups.

**Conclusions:**

The uMAC and SRC had worse and favorable prognosis compared with PDCA, respectively. However, there were no differences in survival by pathological stage, thus histological type was not an independent predictor of prognosis.

## Background

According to the World Health Organization (WHO) classification, the four predominant histological types of gastric adenocarcinoma are tubular adenocarcinoma, papillary adenocarcinoma, mucinous adenocarcinoma (MAC) and signet ring cell carcinoma (SRC) [1]. Unlike tubular adenocarcinoma, which is graded as well-, moderately- or poorly-differentiated according to the degree of glandular formation, papillary adenocarcinoma is usually classified as well-differentiated, and SRC as poorly-differentiated. The Japanese classification system categorizes gastric adenocarcinomas into two groups: differentiated and undifferentiated. The differentiated group consists of well-differentiated, moderately-differentiated and papillary adenocarcinoma. The undifferentiated group consists of poorly differentiated adenocarcinoma (PDAC) and SRC. Interestingly, MAC can be regarded as either a differentiated or undifferentiated type depending on the predominant components [2]. In the same context, Nakamura categorized all gastric cancer as either differentiated or undifferentiated [3,4].

Undifferentiated-type gastric adenocarcinomas in general have a worse prognosis. The innate characteristics and prognosis of MAC and SRC have been studied [5-18]. However, the results of those studies are still debated. Although MAC definitely has a dismal prognosis, several studies have reported that this is due to its typically advanced stage at diagnosis, rather than its cellular nature [17-20]. In addition, the clinicopathological features of SRC remain unclear. Some studies reported that early-stage gastric SRC was associated with a better prognosis than non-SRC, but in advanced gastric cancer, SRC histology was not an independent prognostic factor [6-9]. Others insisted that SRC had a prognosis similar to non-SRC [13,14], or that SRC was an independent predictor of a poor prognosis [15]. However, most studies have compared one type, such as MAC or SRC, with all other types of gastric cancer. Our hypothesis was that a comparison limited to only undifferentiated types would provide a more practical analysis. Moreover, an appropriate standard of comparison is required to clarify the oncologic significance of these cell types. Therefore, in this study, we set PDAC as the comparison object in order to determine the characteristics of particular histologies, such as MAC and SRC.

## Methods

### Definition

In accordance with the WHO [1] and the Japanese Gastric Cancer Association (JGCA) [2], we defined PDAC as tubular adenocarcinoma composed of highly irregular glands that are recognized with difficulty, or single cells that remain isolated or are arranged in small or large clusters. MAC and SRC were defined as tumors in which more than 50% of the tumor area contained extracellular mucin pools and tumors consisting of isolated or small groups of malignant cells containing intracytoplasmic mucin, respectively.

### Patients

Between 1989 and 2005, a total of 2,709 patients diagnosed with gastric cancer underwent surgery at Seoul St. Mary’s Hospital. All of the surgical procedures were performed by three experienced gastric surgeons of our single institution with a definitive treatment guideline for gastric cancer. An eligibility criterion was patients who underwent gastrectomy accompanied by lymph node dissection with a primary gastric adenocarcinoma. Patients with synchronous malignancies or remnant gastric cancer and those who were diagnosed as differentiated-type adenocarcinoma (papillary adenocarcinoma, well and moderately tubular adenocarcinma, and differentiated-type MAC) were excluded from the present study. Finally, 1,002 patients histologically diagnosed as PDAC, 54 diagnosed as undifferentiated-type MAC (uMAC), and 320 diagnosed as SRC were enrolled in the study. Clinicopathological parameters, including gender and age of patients; number, size and location of tumors; depth of invasion; lymph node metastasis status; lymphovascular and perineural invasion; and operative details, were collected retrospectively from the Gastric Cancer Patients Registry of Seoul St. Mary’s Hospital. Cancer staging was as described in the seventh edition of the International Union Against Cancer TNM classification [21].

Regular follow-up programs were conducted; these involved the determination of tumor marker levels, abdominal computed tomography (CT) and endoscopic examination, according to our standard protocol (every 3 and 6 months for advanced and early gastric cancer, respectively, for the first 3 years; and every 12 months thereafter). The mean follow-up period of the enrolled patients was 92.3 ± 68.7 months (range, 0.3 to 267.4 months; *n* = 1,425). Survival rates were repeatedly determined using the registration data of the Korea National Statistical Office (KNSO) and medical records.

Written informed consent was obtained from the patient for publication of this report and any accompanying images. This study was approved by the institutional review board of the ethical committee of the College of Medicine, Catholic University of Korea (KC11RISI0686).

### Statistical analysis

Differences between groups were analyzed using the *t*-test for continuous variables and the χ^2^ test or Fisher’s exact test for proportions. Survival analysis was performed using Kaplan-Meier methods with a log-rank test for univariate analysis, and multivariate analysis for survival was performed using a Cox proportional hazards model with the ‘Backward LR’ method. Statistical analyses were performed with SPSS version 13.0 (SPSS, Inc., Chicago, IL, USA), and *P*-values <0.05 were taken to indicate statistical significance.

## Results

There were no significant differences in surgical procedure, including fulfillment of curative resection, between patients diagnosed with the three histological types (Table 1). In terms of clinicopathological characteristics, uMAC patients were significantly older and had tumors of larger diameter. A diffuse type of Lauren’s classification was most common in SRC patients and intestinal type was not observed. Lymphatic and perineural invasion were significantly lower in SRC than in the other types and vascular invasion was higher in uMAC. In contrast to uMAC tumors, which were present at an advanced stage with deeper invasion and more lymph node involvement, SRC was typically detected at an earlier stage. PDAC tumors tended to be detected at stages between those of uMAC and SRC (Table 2).

**Table 1:** Operative findings

**Variable**	**PDAC**	**uMAC**	**SRC**	***P-*value**
	***n*= 1002**	***n*= 54**	***n*= 320**	
Extent of resection, n (%)				
Subtotal	683 (68.2)	38 (70.4)	230 (71.9)	0.448
Total	319 (31.8)	16 (29.6)	90 (28.1)	
Lymph node dissection, n (%)				
D1	66 (6.6)	6 (11.1)	23 (7.2)	0.167
D1+	54 (5.4)	1 (1.9)	26 (8.1)	
More than D2	882 (88.0)	47 (87.0)	271 (84.7)	
Reconstruction, n (%)				
Billroth-I	95 (9.5)	5 (9.3)	44 (13.8)	0.240
Billroth-II	588 (58.7)	31 (57.4)	186 (58.1)	
Roux-en-Y	319 (31.8)	18 (33.3)	90 (28.1)	
Combined resection, n (%)				
Present	123 (12.3)	8 (14.8)	31 (9.7)	0.356
Absent	879 (87.7)	46 (85.2)	289 (90.3)	
Curability, n (%)				
Curative	876 (87.4)	45 (83.3)	291 (90.9)	0.132
Non-curative	126 (12.6)	9 (16.7)	29 (9.1)	

**Table 2:** Clinicopathological characteristics

**Variable**	**PDAC**	**uMAC**	**SRC**	***P-*value**
	***n*= 1002**	***n*= 54**	***n*= 320**	
Age, years (mean ± SD)	54.6 ± 12.8	57.3 ± 12.1	52.4 ± 12.0	0.003
Gender, n (%)				
Male	605 (60.4)	35 (64.8)	187 (58.4)	0.637
Female	397 (39.6)	19 (35.2)	133 (41.6)	
Multiplicity, n (%)				
Single	982 (98.0)	53 (98.1)	316 (98.8)	0.612
Multiple	20 (2.0)	1 (1.9)	4 (1.3)	
Tumor location, n (%)				
Upper	108 (10.8)	4 (7.4)	22 (6.9)	0.184
Middle	392 (39.1)	17 (31.5)	141 (44.1)	
Lower	452 (45.1)	31 (57.4)	139 (43.4)	
Whole	50 (5.0)	2 (3.7 %)	18 (5.6)	
Tumor size, cm (mean ± SD)	5.7 ± 3.7	7.1 ± 3.8	4.8 ± 3.2	<0.001
Lauren, n (%)				
Intestinal type	0 (0)	0 (0)	0 (0)	<0.001
Diffuse type	782 (78.0)	37 (68.5)	290 (90.6)	
Mixed type	220 (22.0)	17 (31.5)	30 (9.4)	
Lymphatic invasion, n (%)				
Present	607 (60.6)	42 (77.8)	120 (37.5)	<0.001
Absent	395 (39.4)	12 (22.2)	200 (62.5)	
Vascular invasion, n (%)				
Present	105 (10.5)	12 (22.2)	27 (8.4)	0.009
Absent	897 (89.5)	42 (77.8)	293 (91.6)	
Perineural invasion, n (%)				
Present	490 (48.9)	29 (53.7)	108 (33.8)	<0.001
Absent	512 (51.1)	25 (46.3)	212 (66.3)	
Depth of invasion, n (%)				
T1	248 (24.8)	3 (5.6)	164 (51.3)	<0.001
T2	152 (15.2)	4 (7.4)	32 (10.0)	
T3	242 (24.2)	13 (24.1)	40 (12.5)	
T4	360 (35.9)	34 (63.0)	84 (26.3)	
Lymph node metastasis, n (%)				
N0	422 (42.1)	9 (16.7)	201 (62.8)	<0.001
N1	134 (13.4)	11 (20.4)	25 (7.8)	
N2	131 (13.1)	10 (18.5)	27 (8.4)	
N3	315 (31.4)	24 (44.4)	67 (20.9)	
Pathological stage, n (%)				
I	323 (32.2)	4 (7.4)	180 (56.3)	<0.001
II	212 (21.2)	11 (20.4)	50 (15.6)	
III	354 (35.3)	30 (55.6)	64 (20.2)	
IV	113 (11.3)	9 (16.7)	26 (8.1)	

**Figure 1:**
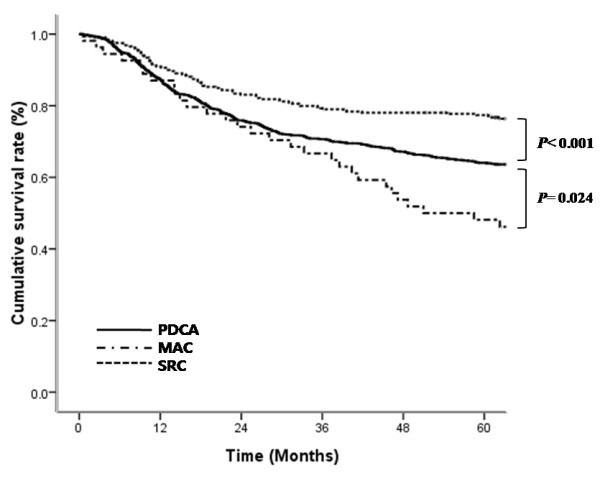
Survival curves of patients with mucinous adenocarcinoma (MAC), signet ring cell carcinoma (SRC), or poorly differentiated adenocarcinoma (PDAC).

**Figure 2:**
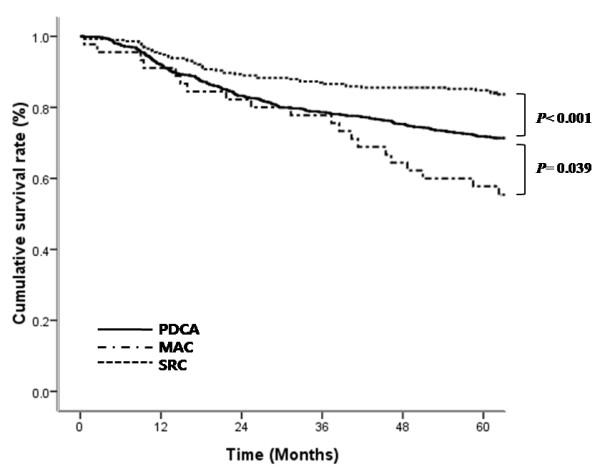
Survival curves for patients with MAC, SRC, or PDAC who underwent curative resection.

There was a significant difference in overall five-year survival between patients with SRC (77.4%) and those with PDAC (64.0%, *P*<0.001) or uMAC (48.1%, *P*<0.001). The five-year survival rates also differed significantly between patients with PDAC and uMAC (*P* = 0.024) (Figure 1). Of the 1,212 patients who underwent curative resection, the overall five-year survival of patients with SRC (84.8%) was significantly higher than that of patients with PDAC (71.9%; *P*<0.001) or uMAC (57.8%; *P*<0.001), and there was significant difference in five-year survival between PDAC and uMAC (*P* = 0.039) (Figure 2).

In a comparison of five-year survival rates in the three histological groups by pathological stage, there were no significant differences among either all or curatively resected patients (Table 3). In addition, no significant difference in the five-year survival rates was detected among any pathological T/N-stage (data not shown).

**Table 3:** Comparison of five-year survival rates according to pathological stage

**Stage**	**PDAC**	**uMAC**	**SRC**	***P-*value**
All, n (%)				
I	95.3	100.0	96.6	0.575
II	77.0	81.8	84.0	0.333
III	45.9	43.3	49.2	0.791
IV	5.5	11.1	3.8	0.421
Curative, n (%)				
I	95.3	100.0	96.6	0.575
II	77.6	81.8	84.0	0.369
III	48.3	44.8	53.4	0.696

Multivariate analysis using a Cox proportional hazard regression model showed that older age, male gender, depth of invasion, lymph node metastasis and curability were independently associated with a poor prognosis (Table 4). Among patients who had undergone curative resection, older age, tumor diameter, depth of invasion and lymph node metastasis were identified as independent prognostic factors (Table 5). Histological type was not an independent prognostic factor in either group.

**Table 4:** Multivariate analysis of prognostic factors for survival in all enrolled patients

**Variable**	**Coefficient**	**SE**	**Hazard ratio (95% CI)**	***P-*value**
Age, years				
≥65/<65	0.546	0.093	1.727 (1.440 to 2.072)	<0.001
Gender				
Male/Female	0.229	0.088	1.257 (1.057 to 1.495)	0.010
Tumor size, cm				
≥5/<5	0.152	0.111	1.164 (0.936 to 1.447)	0.171
Depth of invasion				
T2/T1	0.618	0.204	1.856 (1.245 to 2.766)	0.002
T3/T1	0.856	0.188	2.353 (1.626 to 3.403)	<0.001
T4/T1	1.532	0.184	4.629 (3.227 to 6.641)	<0.001
Lymph node metastasis				
N1/N0	0.077	0.175	1.080 (0.766 to 1.523)	0.661
N2/N0	0.614	0.158	1.847 (1.356 to 2.516)	<0.001
N3/N0	1.100	0.136	3.005 (2.301 to 3.925)	<0.001
Curability				
Non-curative/Curative	1.104	0.110	3.016 (2.433 to 3.740)	<0.001
Histology				
PDAC/SRC	0.071	0.114	1.074 (0.858 to 1.343)	0.534
uMAC/SRC	−0.018	0.205	0.982 (0.658 to 1.467)	0.930

**Table 5:** Multivariate analysis of prognostic factors for survival in enrolled patients who underwent curative resection

**Variable**	**Coefficient**	**SE**	**Hazard ratio (95% CI)**	***P-*value**
Age, years				
≥65/<65	0.667	0.108	1.949 (1.576 to 2.409)	<0.001
Gender				
Male/Female	0.200	0.103	1.221 (0.998 to 1.494)	0.053
Tumor size, cm				
≥5/<5	0.237	0.118	1.268 (1.007 to 1.597)	0.043
Depth of invasion				
T2/T1	0.582	0.208	1.789 (1.190 to 2.689)	0.005
T3/T1	0.749	0.196	2.114 (1.439 to 3.106)	<0.001
T4/T1	1.433	0.196	4.192 (2.855 to 6.155)	<0.001
Lymph node metastasis				
N1/N0	0.015	0.192	1.016 (0.696 to 1.481)	0.936
N2/N0	0.558	0.168	1.747 (1.256 to 2.428)	0.001
N3/N0	1.161	0.143	3.195 (2.414 to 4.228)	<0.001
Histology				
PDAC/SRC	0.157	0.136	1.170 (0.896 to 1.528)	0.248
uMAC/SRC	0.135	0.242	1.144 (0.712 to 1.840)	0.578

Of the 1,212 patients who underwent curative resection, 290 (23.9%) experienced a recurrence. There was a significant difference among the three groups (*P*<0.001), with uMAC and SRC patients having the highest and lowest recurrence rates, respectively. However, there was no significant difference in recurrence rates among the groups according to pathological stage (stage I, *P* = 0.626; stage II, *P* = 530; stage III, *P* = 0.574). In addition, there were no significant differences in the pattern of recurrence (*P* = 0.819) (Table 6).

**Table 6:** Recurrence rates and patterns

**Variable**	**PDAC**	**uMAC**	**SRC**	***P-*value**
	***n*= 876**	***n*= 45**	***n*= 291**	
Recurrence, n (%)	228 (26.0)	20 (44.4)	42 (14.4)	<0.001
Pattern, n (%)				
Peritoneal	100 (43.9)	7 (35.0)	15 (35.7)	0.819
Lymphatic	34 (14.9)	3 (15.0)	9 (21.4)	
Remnant stomach	15 (6.6)	2 (10.0)	1 (2.4)	
Hematogenous	37 (16.2)	4 (20.0)	9 (21.4)	
Combined	42 (18.4)	4 (20.0)	8 (19.0)	

## Discussion

Undifferentiated types of gastric adenocarcinoma are correlated with aggressive neoplasms associated with more extensive and infiltrative growth, lymph node metastasis and distant metastasis characterized by peritoneal dissemination and, therefore, have a worse prognosis than differentiated types [22,23]. Prognosis for three gastric adenocarcinomas, such as SRC, PDAC and uMAC, which belong to the undifferentiated type, is still not established and there were few studies to deal with only undifferentiated-type gastric adenocarcinomas. For the design of this study, MAC was sub-classified into differentiated-type MAC and uMAC. By our results, SRC and uMAC had the best and worst prognosis, respectively, in a univariate analysis of overall survival. The same trend in survival differences was observed in curatively resected patients. Such a result for uMAC agreed with the previous reports stating that MAC had a dismal prognosis [16-20].

In contrast to MAC, which has shown consistent results, data regarding the prognosis of SRC are conflicting, although most studies did not take into account the differentiation category. Only one report by Fang *et al*. [5] compared SRC and MAC, both of which are mucin-producing gastric cancers. They reported that patients with SRC had better five-year survival than those with MAC in stages I and II gastric cancer, but there was no difference in five-year survival in advanced-stage cancer. However, histological type was not an independent prognostic factor in their multivariate analysis. In the present study, while there was a significantly better five-year survival rate of SRC in all enrolled patients, patients with SRC did not have a more favorable five-year survival than those with uMAC and PDAC according to pathological stage, and histological type was also not associated with prognosis. Thus, the higher proportion of early stage SRC led to its better prognosis. The uMAC also did not have a significantly worse five-year survival rate than the other two types in comparison with each pathological stage. Similarly, an advanced stage of uMAC at diagnosis can explain that cause. Therefore, follow-up and postoperative strategy, such as adjuvant chemotherapy for undifferentiated-type gastric adenocarcinoma, would be tailored based on each pathological stage, irrespective of histological type.

It is uncertain why SRC and MAC present an early and advanced stage at diagnosis, respectively. One possible explanation regarding SRC is that SRC tends to expand superficially to mucosal and submucosal layers. Then, the broad tumor causes the clinical symptoms, such as epigastric soreness, and can be detected at an early stage [24]. With regard to MAC, the role of extracellular mucin has been reported. Extracellular mucin of MAC acts as an infiltrating medium and promotes the dispersion and invasion of tumor cells to deeper layers [10,25]. In addition, mucin can inhibit the inflammatory and immunologic reactions of tumor cells [26].

Although it is known that undifferentiated-type gastric adenocarcinoma is associated with the diffuse type of Lauren classification, few studies have reported a correlation between histology type and Lauren classification. The WHO defined the diffuse type as a histological form comprised of poorly cohesive cells with little or no gland formation [1], and this shape almost matched up with the SRC type. The present study, in which the proportion of the diffuse type was highest in SRC (90.6%), followed by PDAC (78.0%) and uMAC (68.5%), reflected such an association. The intestinal type of undifferentiated-type gastric adenocarcinoma was very rare, and there was no case of the intestinal type in the present study. The structure of the intestinal type was partially observed at the advancing margin of undifferentiated-type gastric adenocarcinoma belonging to the mixed type.

In the present study, a Cox proportional hazard regression model identified older age, male gender, depth of invasion, lymph node metastasis and curability as independent prognostic factors in undifferentiated gastric adenocarcinomas. The N1 stage did not have prognostic significance, and the hazard ratio of the N2 stage was low. The current study was based on the seventh edition of the International Union Against Cancer TNM classification, which has a narrow division of N1 and N2 stages: N1 for one to two positive lymph nodes and pN2 for three to six. Our previous study [27] reported the low power of discrimination of the seventh edition N classification owing to this narrow range. The current N stage results in undifferentiated type cancers were also considered to be attributable to the seventh edition N classification.

This study has a limitation. The survival benefits of adjuvant chemotherapy were not clarified. Most patients diagnosed as having advanced stage received adjuvant chemotherapy, most commonly 5-FU or cisplatin-based systemic chemotherapy, based on our institute’s guidelines. However, this study lacked consistency regarding patient and drug selection because of the nature of very long-term data, especially for second-line chemotherapy.

## Conclusions

Compared with patients with PDAC, the overall survival of patients with uMAC was significantly worse and those with SRC had a better prognosis. However, since there were no differences in five-year survival between histological type according to cancer stage and having no histological type was not an independent prognostic factor, treatment strategy would be focused on the stage of undifferentiated-type gastric adenocarcinoma at diagnosis.

## Abbreviations

WHO: World health organization; MAC: Mucinous adenocarcinoma; SRC: Signet ring cell carcinoma; PDCA: Poorly differentiated adenocarcinoma; JGCA: Japanese gastric cancer association; uMAC: Undifferentiated-type mucinous adenocarcinoma; CT: Computed tomography; KNSO: Korea national statistical office.

## Competing interests

The authors declare that they have no competing interests.

## Authors' contributions

HHLcontributed to study conception and design, analysis of data, drafting of the manuscript and critical revision. KYS was responsible for acquisition and interpretation of data. CHP was responsible for interpretation of data and critical revision. HMJ contributed to study conception and design and critical revision. All authors read and approved the final manuscript.
